# Medical Students’ Perceptions of Clinical Teachers as Role Model

**DOI:** 10.1371/journal.pone.0150478

**Published:** 2016-03-09

**Authors:** Sonia Ijaz Haider, David R. J. Snead, Muhammad Furqan Bari

**Affiliations:** 1 Department for Educational Development, Faculty of Health Sciences, Aga Khan University, Karachi, Pakistan; 2 Department of Pathology, University Hospitals Coventry and Warwickshire NHS Trust, Coventry, United Kingdom; 3 Department of Pathology, Dow International Medical College, Dow University of Health Sciences, Karachi, Pakistan; National Institute of Health, ITALY

## Abstract

**Introduction:**

Role models facilitate student learning and assists in the development of professional identity. However, social organization and cultural values influence the choice of role models. Considering that the social organization and cultural values in South East Asia are different from other countries, it is important to know whether this affects the characteristics medical students look for in their role models in these societies.

**Methods:**

A 32 item questionnaire was developed and self-administered to undergraduate medical students. Participants rated the characteristics on a three point scale (0 = not important, 1 = mildly important, 2 = very important). One way ANOVA and student's t-test were used to compare the groups.

**Results:**

A total of 349 (65.23%) distributed questionnaires were returned. The highest ranked themes were teaching and facilitating learning, patient care and continuing professional development followed by communication and professionalism. Safe environment and guiding personal and professional development was indicated least important. Differences were also observed between scores obtained by males and females.

**Conclusion:**

Globally there are attributes which are perceived as essential for role models, while others are considered desirable. An understanding of the attributes which are essential and desirable for role models can help medical educators devise strategies which can reinforce those attributes within their institutions.

## Introduction

Role modeling has been defined as the “process in which faculty members demonstrate clinical skills, model and articulate thought processes and manifest positive professional characteristics”[1]. Role models are an essential component of medical education because they facilitate student learning and assist in the development of professional identity through the observation of their clinical tutors [2, 3]–in the way they behave and interact with patients, colleagues and others. In a recent study, Burgess *et al*[4], concluded that role models played a critical role in influencing students’ motivation and the behavior they engaged in. Similarly, Byszewski *et al* [5] reported that medical students indicate professionalism is learnt best through role models. Curry *et al* [6] identified teamwork, collegiality and respect as exemplary behaviors which facilitate learning in operation theatres.

Social organization and cultural values also influence the choice of role models [7–9]. McLean[9] conducted a study in which students perceptions regarding role models in a South African medical school were explored. They reported that for students, role models were those who had struggled and succeeded within family or national context, accentuating that cultural background influences students’ choice of role models. In the subsequent study Mclean *et al* [8] further established that identifying a role model from similar origins is important for students to reaffirm their own culture and can also influence their patient interactions. Similarly, Wright and Carresse [7] examined issues related to physicians serving as role models for diverse medical learners and concluded that learners prefer role models who resemble them culturally and find them easier to accept as role model. Passi [10] reviewed existing evidence on role modeling in medical education from January 1990 to February 2012. They reviewed 39 articles and concluded that the majority of the studies were performed in the United States and the remainder in Europe, Australia and some in United Arab Emirates; however no studies were reported from South East Asia. Considering that the social organization and cultural values in South East Asia are different from other countries, it is important to understand if the characteristics of medical students in these countries differ greatly from the existing evidence.

In South East Asia, Pakistan has a number of medical universities and colleges across the country [11]. Some of these universities and colleges are funded by the government while others are private colleges/universities [11]. It is mandatory for all the medical universities and colleges to be recognized by the Pakistan Medical and Dental Council (PM&DC)[12]. PM&DC is a recognized statutory regulatory authority in Pakistan which ensures standards of excellence in medical education are maintained in all the medical institutions. It maintains a list of all the recognized medical institutions and their graduates in Pakistan [12].

The objective of the present study was to identify the characteristics of role models used by medical students studying in a single institute in Pakistan, and to examine similarities and differences between these undergraduates in comparison to available published data for western students. Similarities and differences between male and female respondents are also reported.

## Methods

### Setting and subjects

The study was conducted in the Shalamar Medical and Dental College, University of Health Science in Lahore, Pakistan. The ethical approval for this study was obtained by the Institutional Review Board (IRB) of Shalamar Medical and Dental College in Lahore, Pakistan. The course is taught in English. The undergraduate medical program is a five years degree program in which the medical curriculum integrates Basic and Clinical Sciences. At the time of study, the medical school had 166 full time faculty members. Participants (n = 535) for this study were allunder graduate medical students from year one to year five for the academic year 2013–2014.

### Questionnaire development

For the purpose of this study, a role model is defined as “a person considered as a standard of excellence to be imitated” [13]. A 32 item questionnaire was developed based on the framework for Professional Development of Postgraduate Medical Supervisors [14]. The items were grouped under same headings as given in the framework; continuing professional development as an educator; guiding students in their personal and professional development; patient care; safe environment; teaching and facilitating learning. Items were established to analyze role models, therefore based on a literature review, some of the items pertaining to communication skills and professionalism were also added [15, 16].

Participants were asked to rate these characteristics on a three point scale (0 = least important, 1 = moderately important, 2 = very important). Participants were also asked to list any additional characteristics that they looked for in role models, which were not included in the questionnaire. Items were reviewed by ten faculty members for clarity and pretested on five students who had recently graduated to avoid ambiguity items. Items for the seven themes were randomly arranged in the questionnaire.

The questionnaires were self-administered and collected towards the end of academic year 2014 (from August till October). An introductory letter outlining the purpose of the study, consent form and a written copy of the questionnaire was distributed individually. Participation in the study was voluntary. A written consent was obtained for participating in the study. Questionnaires were completed anonymous and confidentiality of the data was maintained.

### Data Analysis

Mean and standard deviation of scores from all individual items representing each of the seven themes were calculated separately for male and female medical students of each year (year 1 to 5). Differences in the scores achieved by each group were calculated using student's t-test for two groups and ANOVA for multiple group comparisons. P-value of <0.05 was considered significant. All statistical analysis was conducted in SPSS version 20 and Microsoft Excel.

## Results

### Demographics and response rate

A total of 535 questionnaires were distributed. Of these 349 (65.23%) completely filled questionnaires were returned. First year students returned 114 out of 150 (76%), second year students returned 71 out of 100 (71%), third year students returned 46 out of 100 (46%), fourth year returned 71 out of 96 (73.95%) and finally fifth year returned 47 out of the 89 (52.80%) distributed questionnaires. Among 349 returned questionnaires there were 103 (29.51%) male and 246 (70.49%) female respondents.

### Overall ranking of role model themes

The highest ranked themes (most important) by majority of students of all classes irrespective of gender based on the mean of scores for each theme by all students were teaching and facilitating learning (1.66±0.493), patient care (1.628±0.335) and continuing professional development as an educator (1.608±0.335). These were followed by communication skills (1.587±0.347) and professionalism (1.577±0.332) which were considered moderately important. Lastly, guiding personal and professional development (1.571±0.364) and safe environment (1.557±0.321) were considered least important. Differences in ranking of role model themes by year one to year five students irrespective of gender are summarized in Table 1.

**Table 1:** Differences in ranking of role model characteristics by different classes of students.

	First year students	Second year students	Third year students	Fourth year students	Fifth year students
**Rank 1**	Teaching and facilitating learning	Teaching and facilitating learning	Communicator	Teaching and facilitating learning	Teaching and facilitating learning
**Rank 2**	Patient care	Guiding personal and professional development	Patient care	Patient care	Patient care
**Rank 3**	Continuing professional development as an educator	Communicator	Continuing professional development as an educator	Continuing professional development as an educator	Continuing professional development as an educator
**Rank 4**	Professionalism	Continuing professional development as an educator	Professionalism	Safe environment	Communicator
**Rank 5**	Guiding personal and professional development	Patient care	Teaching and facilitating learning	Communicator	Guiding personal and professional development
**Rank 6**	Communicator	Safe environment	Safe environment	Professionalism	Professional
**Rank 7**	Safe environment	Professionalism	Guiding personal and professional development	Guiding personal and professional development	Safe environment

### Pair wise comparison of rating scores among different year students

The majority (>57%) of statistically significant differences were observed between first and fourth year students in the themes a) Teaching and facilitating learning; b) Professionalism; c) Patient care and d) Guiding personal and professional development. Followed by third and fourth year students where statistically significant differences were observed in two themes; a) Communicator and b) Professionalism. Lastly, fourth and final year students rating scores differed significantly in the theme professionalism. Mean ratings of scores and p-values are summarized in Table 2.

**Table 2:** Pair wise comparison of rating scores among different year students.

Themes	Year 1 n = 114(mean ± S.D.)	Year 2 n = 71(mean ± S.D.)	Year 3 n = 46(mean ± S.D).	Year 4 n = 71(mean ± S.D.)	Year 5 n = 47(mean ± S.D.)	p-values
					
Communicator	1.572±0.341	1.611±0.283	1.648±0.334*	1.497±0.379*	1.664±0.391	*0.009
Continuing professional development as an educator	1.606±0.312	1.603±0.296	1.643±0.368	1.549±0.368	1.674±0.355	NS
Guiding personal and professional development	1.579±0.315*	1.625±0.324‡	1.552±0.439	1.459±0.373*‡	1.655±0.41	*0.01‡0.008
Patient care	1.667±0.301*	1.575±0.312	1.643±0.37	1.559±0.338*	1.699±0.389	*0.01
Safe environment	1.561±0.298	1.556±0.272	1.579±0.349	1.513±0.319	1.598±0.414	NS
Professionalism	1.601±0.291*	1.554±0.32	1.641±0.337‡	1.477±0.343*‡†	1.645±0.391†	*0.005‡0.006†0.02
Teaching and facilitating learning	1.69±0.463†	1.63±0.514	1.63±0.532	1.58±0.497†	1.77±0.476	†0.04

*‡† indicates statistically significant differences in pair wise comparison among two classes; NS = No statistically significant difference observed in any pair wise comparison; S.D. = Standard deviation

### Group comparison of rating scores among male and female students

Except for the theme "Professionalism", ranking scores of all themes between male and female students were statistically different (p<0.05). Mean ratings of scores and p-values are summarized in Table 3

**Table 3:** Group comparison of male and female students.

Themes	Male n = 103(mean ± S.D.)	Female n = 246(mean ± S.D.)	p-value
Communicator	1.488±0.402	1.628±0.313	0.001
Continuing professional development as an educator	1.529±0.406	1.641±0.294	0.005
Guiding personal and professional development	1.499±0.403	1.601±0.343	0.005
Patient care	1.538±0.366	1.665±0.315	0.0005
Safe environment	1.477±0.383	1.591±0.286	0.003
Professionalism	1.532±0.357	1.596±0.32	NS
Teaching and facilitating learning	1.5±0.54	1.73±0.455	0.0004

NS = No statistically significant difference observed in group comparison; S.D. = Standard deviation

## Discussion

According to Wenger’s theory of communities of practice, participation of peripheral members in a community of practice depends on the core members, whose facilitation and supervision enhances learning [17]. In undergraduate medicine a major portion of teaching is in the real clinical settings [18]. Teachers, i.e. the medical school community core members, according to Wengers theory [17], play a pivotal role in facilitating students, the peripheral members, by providing them with facilitated learning and supervision in the clinic [19]. In the present study teaching and facilitating learning is identified as one of the top attributes of role models, consistent with Wenger’s theory. Other studies [4, 20, 21] have also corroborated this finding; however in the present study this is identified as one of the highest rank attribute which is different compared to existing evidence.

In accordance with western studies [1, 4, 13, 20, 22–25], this study also showed that students ranked patient care as one of top (second highest) attributes of role models. The essence of medical education is to ensure that best practices are followed in patient care and these results show that even early pre-clinical students recognize the value of optimal patient care in seeking role models. Good clinical practice is regarded as important despite examples in which quality of care is compromised because of lack of resources in Pakistan. A plausible reason why the majority of the students emphasized it most important compared to other characteristics.

In the present study, continuing professional development as an educator has been ranked as one of the top three attributes by both male and female students. Other studies [26, 27] reported that positive role models continually update their knowledge and skills, engage in self-reflection and self-assessment. It is essential [28] that clinical teachers should continue to engage in professional practice, update their knowledge and maintain development of their clinical practice, but medical students preferring it as one of the important attributes implies recognition amongst students of the importance that professional development plays in their chosen role models. Findings from the present study imply that students recognize that clinical teachers are performing dual jobs, acting both as a clinician and teacher. Unless protected time is provided, continued professional development is often neglected [29].

This study identified that students recognize communication as one of the important characteristics for a role model. In previous studies [6, 15] patient communication has been identified as one of the important attributes of role models. This may imply that medial students recognize that as role models clinical teachers should be able to communicate effectively both as a clinician and as a teacher. Pakistan is a country in which different languages and dialects prevail, and this leads to challenges in communication, especially concerning patients from rural areas.

Professionalism was also indicated as one of the important characteristic of role models. This concurs with other studies in which positive role models demonstrate humanistic values [5, 30, 31]. In one study respect, integrity and honesty were highly ranked (28). Although the prevalent culture values respect, care, compassion, integrity and honesty, it is possible in pressurized situations with time a resource constraints, some aspects of a professional approach may be compromised our study shows an ability to maintain professional standards is seen as an important attribute in role models. This is supported by other studies in which emphasis is on creating and promoting a professional environment [32, 33].

A safe environment was regarded to be of low important characteristics of role models. These findings are in congruence with other studies [18, 34] where creating positive learning environment facilitates learning. Although this variance from the previous literature could be due to cultural differences, there are other reasons which might explain this, for example the medical school promotes an overall positive learning environment, in which mutual respect between students and teachers is promoted. Students therefore are already supported in articulating their opinions about different issues and collegiality promotes teamwork and cooperation for patient care. Similarly guiding personal and professional development was rated least important. Again apart from cultural differences other plausible reasons could be that the institution offers continuous counseling, career and support services to students in all years. In addition there is a mentoring programming and all students have a mentor with whom they meet regularly to discuss their academic and personal development.

Overall findings from the present study indicate that students perceived role models should exhibit the following attributes: patient care, teaching and learning, continuing their professional development, communication skills and professionalism. There were certain differences in the ranking of year 2 and 3 students. A plausible explanation could be that in year 3, students commence their clinical rotations on the wards and experience for the first time patient care, management and challenges. Therefore for them patient care, effective communication and continuous development as a professional is important compared to year 2 students for whom teaching and learning, guiding personal and professional development and communication skills are important. Although the ranking of these attributes differed, these findings are broadly in line with existing evidence. Safe environment and guiding personal and professional development were considered less important in our study it is unclear if this is due to cultural differences or due to differences in the way medical students are supported at our institution[35].

The collective response rate of the questionnaires was fairly adequate however year three and year five had the lowest percentage of respondents. One of the reasons could be administration of questionnaires towards the end of the academic year, during which year three students were completing their clinical rotations in different clinical disciplines while year five students were preparing for their annual examinations. The rationale for administering the questionnaires towards the end of the academic year was to ensure that students become familiar with all the teachers to facilitate them in answering the questionnaire. However in retrospect, if the questionnaires were administered to year three and five in the middle of the academic year, a better response rate could have been possible.

This study is limited to a single institution, however considering that the culture and values are similar across Pakistan, we believe that our findings are broadly representative of students across Pakistan. The study did not examine what the role models believe are important role models or how they perceive local culture to affect role models. Exploring how clinical teachers perceive themselves as role models and the values they prefer the students to learn may be useful in future studies.

## Conclusion

In conclusion, the preferred characteristics of role models in Pakistan medical students are similar to other reported studies. Students in the undergraduate medical program of Pakistan indicated teaching and facilitating learning, patient care, and continuing professional development as very important. Communication and professionalism were considered mildly important, while safe environment and guiding personal and professional development were indicated least important. An understanding of the attributes which are essential and desirable for role models can help medical educators devise strategies which can reinforce those attributes within their institutions.
